# EMIRGE: reconstruction of full-length ribosomal genes from microbial community short read sequencing data

**DOI:** 10.1186/gb-2011-12-5-r44

**Published:** 2011-05-19

**Authors:** Christopher S Miller, Brett J Baker, Brian C Thomas, Steven W Singer, Jillian F Banfield

**Affiliations:** 1 Department of Earth and Planetary Science, University of California, Berkeley, 307 McCone Hall #4767, Berkeley, CA 94720, USA; 2 Current address: Department of Geological Sciences, University of Michigan, 1100 N. University Ave, Ann Arbor, MI 48109, USA; 3 Earth Sciences Division, Lawrence Berkeley National Laboratory, 1 Cyclotron Road, Mail Stop 90-R1116, Berkeley, CA 94720, USA; 4 Deconstruction Division, Joint BioEnergy Institute, 5885 Hollis St, Emeryville, CA 94660, USA; 5 Department of Environmental Science, Policy and Management, University of California, Berkeley, 336 Hilgard Hall, Berkeley, CA 94720, USA

## Abstract

Recovery of ribosomal small subunit genes by assembly of short read community DNA sequence data generally fails, making taxonomic characterization difficult. Here, we solve this problem with a novel iterative method, based on the expectation maximization algorithm, that reconstructs full-length small subunit gene sequences and provides estimates of relative taxon abundances. We apply the method to natural and simulated microbial communities, and correctly recover community structure from known and previously unreported rRNA gene sequences. An implementation of the method is freely available at https://github.com/csmiller/EMIRGE.

## Background

Characterization of microbial community composition is most often done with a phylogenetic marker gene, most commonly the small subunit ribosomal RNA (SSU rRNA) gene [1]. Traditionally, rRNA sequences were generated by amplification, cloning, and Sanger sequencing. More recently, technologies such as pyrotag sequencing of short hyper-variable regions [2,3], Illumina sequencing of variable tags [4-7], and hybridization to specialized high-density microarrays (for example, Phylochip) [8-10] have accelerated the throughput of SSU-based microbial community characterization. Although each method has limitations, these high-throughput approaches have been broadly adopted, and have provided new understanding of microbial community composition from a wide range of environments[8,11,12]. Complementing these approaches are growing databases of SSU sequences from both isolates and environmental samples [13-15] that provide a rich phylogenetic and ecological context.

Searching for SSU genes directly in metagenomic data avoids PCR and primer biases [16,17]. For example, novel deeply branching archaea with unusual 16S rRNA gene sequences were recently detected through metagenomic sequencing [18]. These divergent sequences were not recovered by methods that relied on amplification with standard SSU primers.

Most reported metagenomic sequencing has used Sanger or Roche 454 sequencing technologies. The rRNA gene sequences for closely related organisms in these datasets co-assemble. The result is a composite sequence that is not representative of any community member and obscures the real level of diversity. These problems are exacerbated when shorter sequencing reads are used. Typical reads from the Illumina platform currently range from 35 to 125 bp. Additionally, the k-mer-based methods used to assemble short read data can further confound *de novo *assembly near regions with high inter-species sequence identity, such as that found within the SSU gene. Because of these challenges, there are no methods currently available to assemble full-length SSU sequences from short read sequencing data.

An alternative to *de novo *assembly of short read sequencing data is to map reads to a reference sequence, if one is available. Several fast, memory-efficient mapping programs are available, all of which allow for varying levels of error while searching for alignments [19-21]. Quantification of species abundance could in theory reduce to a read mapping problem if the community composition was known ahead of time and all reference SSU sequences were available. However, the composition of environmental samples is rarely known ahead of time. Short read lengths and high conservation of sequence in the SSU gene produce ambiguous assignments of many reads among closely related strains, confounding a simple mapping strategy.

Here, we report a novel iterative mapping method, based on the expectation maximization (EM) algorithm [22], that accurately reconstructs the full-length SSU sequences present in a microbial community. The method, referred to as expectation maximization iterative reconstruction of genes from the environment (EMIRGE), takes as inputs the raw reads and quality values from a short-read DNA sequencing project and an initial large database of curated SSU sequences. Several iterative read-mapping cycles are completed, during which the most probable consensus sequences are gradually discovered and corrected by the mapped reads. The algorithm produces probabilistically described full-length SSU sequences, and a measure of their relative abundances in the community. This bioinformatic approach can be applied to both shallow and deeply sampled microbial communities with widely varying complexity.

## Results

### *De novo *assembly of microbial communities fails to recover SSU genes

The study used data from one natural and two simulated communities. The natural community, a microbial biofilm containing eukaryotes, bacteria, archaea, and viruses, was sampled from an acid mine drainage site within the Richmond Mine at Iron Mountain, California [23]. Microbial biofilms from this system have been studied extensively as model communities, and 12 near-complete genomes have been assembled from community genomic datasets [24-26]. In the current study, we assembled one lane of 76-bp paired end Illumina sequence (approximately 38.6 million reads, 2.9 Gbp of sequence) and attempted to recover full-length rRNA genes.

For the two simulated communities, we first reconstructed *in silico *a recently described 'simple' model microbial community used by Morgan *et al*. [27] to evaluate DNA extraction and sequencing methods. This community contains eight bacterial species, one archaeon, and one yeast, representing both closely and distantly related taxa that broadly cover the tree of life at various levels of relatedness. We also simulated data from a 'complex' human gut mock community of known composition described by Turnbaugh *et al *[28]. The complex community consisted of 67 taxa with relative abundances ranging over five orders of magnitude. We generated random reads for both simulated communities and applied to these data error profiles (quality values) sampled from the natural community data (see Materials and methods).

**Figure 1:**
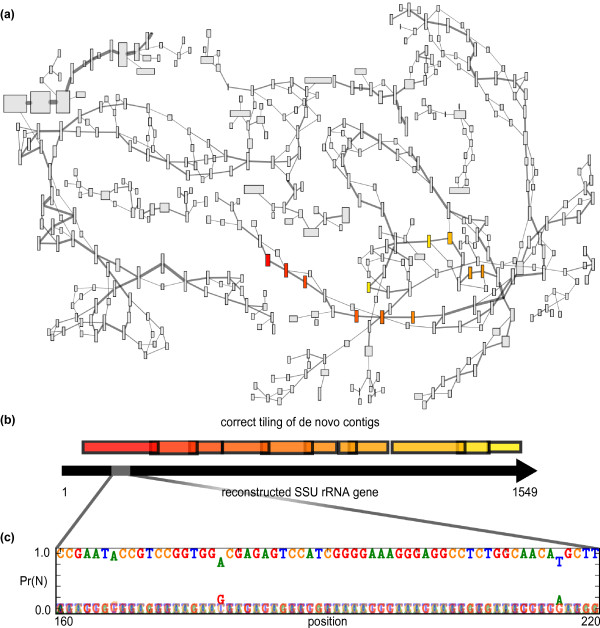
***De novo *assembly of SSU rRNA genes versus reconstruction of full-length gene sequences**. **(a) **A section of the de Bruijn graph created by the short read assembler Velvet [29] for the natural microbial community. Each contig in the graph is represented by a rectangle whose width is proportional to contig length and whose height is proportional to contig k-mer coverage depth. Edge width reflects the multiplicity of overlapping k-mers shared by contigs. All contigs with BLAST matches to SSU genes recovered by EMIRGE were selected, and those contigs and additional contigs within three edges are shown. Contigs with BLAST matches to the SSU sequence from *Leptospirillum ferrodiazotrophum *[54] are shown in color. **(b) **The correct tiling of highlighted contigs from (a) is shown schematically with the EMIRGE-reconstructed SSU rRNA gene. **(c) **A selected region of the *L. ferrodiazotrophum *SSU gene shows the individual base probabilities at algorithm termination for each position in the reconstructed SSU gene. While most bases are highly confident, some positions show evidence for strain variants present in the population.

Assemblies used Velvet, a program developed to assemble short read data [29]. The assembly for the natural community had an N50 of 3,912 nucleotides (half of the assembled length was in contigs of 3,912 nucleotides or longer; data not shown). However, the only near-full-length SSU gene recovered was that of a dominant, near-clonal fungal species. The N50 of bacterial and archaeal SSU fragments with a reliable blast hit to the Silva ribosomal SSU database [15], in contrast, was only 182 bp. Similarly short SSU fragments were recovered from assemblies of both simulated datasets (Table 1). Velvet produces a de Bruijn graph that provides an overview of the assembly. In the vicinity of contigs sampling the 16S rRNA genes, the graph shows a convoluted network of short contigs (nodes) with highly variable coverage (Figure 1).

**Table 1:** Comparison of EMIRGE with *de novo *assembly

Method	Community	Number of expected SSUs	Number of SSU sequences or fragments	Number classified at genus level^a^	N50	Weighted UniFrac distance
EMIRGE	Simulated simple	10	11	10 (90%)	1,537	0.03
Assembly fragments	Simulated simple	10	78	39 (50%)	383	0.32
						
EMIRGE	Simulated complex	52^b^	23	20 (87%)	1,488	0.04
Assembly fragments	Simulated complex	52^b^	320	212 (66%)	122	0.26
						
EMIRGE	Natural	Unknown	11	9 (82%)	1,484	NA
Assembly fragments	Natural	Unknown	137	85 (62%)	182	0.32^c^

^a^RDP classifier with bootstrap cutoff > 50%; eukaryotes not classified. ^b^With abundance > 0.5%. ^c^Distance to EMIRGE-defined community. NA, Not Applicable: true community structure is unknown

### EMIRGE overview: iterative mapping and correction of reference SSU sequences

**Figure 2:**
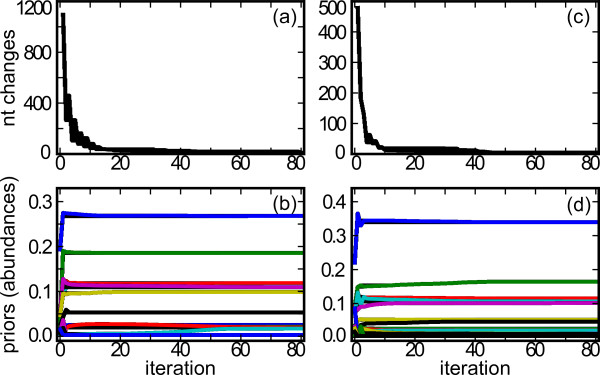
**Convergence of reconstructed SSU sequences and abundance estimates**. **(a-d) **Algorithm convergence for both the simulated simple microbial community (a, b) and natural community (c, d) is shown. In (a, c), the number of nucleotide (nt) changes made in all reconstructed SSU sequences is plotted for each iteration. In (c, d), each line represents a different reconstructed SSU sequence: the prior probability (abundance estimate) of each SSU sequence is plotted for each iteration. Only SSU sequences with ≥ 1% prior probability at convergence are shown.

As full-length rRNA genes could not be recovered from the *de novo *assemblies, we developed a strategy based on mapping of all reads to a large reference database of SSU sequences and iterative determination of the most probable full gene sequences. We chose as a reference database a filtered subset of the SSU sequences contained in Silva [15]. An ideal mapping strategy would not depend on the completeness or correctness of the reference database, and evaluate the probabilities of errors in the mapping and the sequence of the reads. Therefore, we developed a method that models reads as being generated by unknown reference SSU sequences. Each iteration consists of mapping reads to the current reference sequences, computing the probability that each read was generated by each reference sequence, computing estimates of reference sequence abundance, and then correcting the nucleotides of the reference sequences before the subsequent iteration and mapping begins anew. Gradually, the correct reference sequences and the estimates of organism abundance adjust and then stabilize, at which point the iterations stop (Figure 2). At each iteration, if two reference sequences have evolved to be close to identical, we merge them. Conversely, if the evidence from mapping indicates separate strains mapping onto the same reference, the reference is split into two sequences for future iterations.

Central to our approach to determining both the correct SSU gene sequence and computing abundances of each sequence type is a probabilistic approach that acknowledges that we do not know which reference sequence each read should be mapped to. Especially for communities with many closely related strains, and because of sequencing errors, many assignments of reads to specific reference sequences are uncertain. There is even more uncertainty if the read was generated by a sequence not represented in the reference database. The expectation maximization algorithm [22] provides a computational framework for explicitly dealing with this uncertainty. The actual sequences present in the community are unlikely to be present in the SSU database. Thus, the SSU database sequences serve as candidate initial sequences and the probability that each base is correct changes with each iteration. For each mapping cycle and final sequence determination, the base with the highest probability is chosen at each position.

We use the EM algorithm to alternate between an expectation step (E-step), in which the probability for each read being generated by each reference sequence is computed, and a maximization step (M-step), in which we calculate both (i) the probability values for each base in each reference and (ii) a prior probability that each candidate reference generated any read.

### The E-step: computing the probability that a SSU sequence generates a read

In the E-step we construct a distribution representing the probability that each reference sequence generated each read of interest. To compute the probability that a specific reference sequence *s *generated the observed read *r*, Pr*(s|r)*, we use Bayes' theorem:

### The E-step likelihood

To calculate the likelihood, Pr*(s|r)*, for a given read-reference pair, we make the false but simplifying assumption that each base position in the read is independent. The probability that the reference SSU sequence generated the read is thus a product over all mapped positions (*k*) of the probabilities of observing each base *b_k_*, given the reference SSU sequence:

To compute Pr*(b_k_|s)*, we consider the possibility that the reference base *n *can be one of A, C, T, or G:

The first term in the summation is the probability of observing read base *b_k_*, given that the reference sequence base at position *k *is *n*, and can be calculated with the following:

where *p_k _*is the error probability of the called base *b_k _*in the read, as reported by the base-calling software. We make the simplifying assumption that a base mismatch has an equal probability of being one of the three non-match bases. The indicator variable *I *is one if and only if the base called in the read matches the mapped base in the reference sequence. The second term in the summation above, Pr(*n*), is calculated based on the current alignment of reads to the reference sequence from the previous M-step in the EM algorithm (see below).

#### The E-step prior and normalization factor

The prior, Pr(*s*), is the current best estimate of the abundance ratios of each underlying SSU reference sequence in the microbial community. This is computed in the previous M-step (see below). The denominator of Bayes' theorem is calculated in a similar fashion as the likelihood and prior, except that all possible reference SSU sequences (*s_i_) *are considered in the summation. Practically, this reduces to the set of SSU sequences with any reported mappings for the read under consideration: we treat all other sequences as having zero probability.

### The M-step: computing SSU sequence and abundance probabilities

In the M-step, the model parameters representing the candidate reference sequences and their abundances are updated, based on the current best estimate of Pr*(s|r) *from the previous E-step.

#### Correction of the reference sequence

We can calculate Pr*(n) *for each base position in each reference sequence. Ignoring sequence quality scores and with each read mapped to only one sequence, a maximum likelihood estimator for the probability of, for example, base A at reference sequence position *k *would simply be a summation of all A bases in reads mapped at that position divided by the total number of reads mapped at that position. However, because the generating reference sequence for each read is unknown, we instead compute the probability of that base at position *k *based on the current calculated Pr*(s|r) *from the previous E-step, as well as quality scores of mapped bases:

where *b_k, j _*is the aligned base at position *k *in read *j, I *is an indicator variable indicating a match of the aligned base with the base under consideration, *n*, and *P_k, J _*is the error probability of the aligned base *k *in read *j*. The consensus sequence chosen for the next round of mapping is simply the sequence of bases with the highest probability at each position (Figure 1c).

#### Adjusting reference sequence abundances

In each M-step, we also calculate the prior probabilities (abundances) of each reference sequence, based on the current calculated Pr*(s|r) *from the previous E-step. Again, if the reference sequence generating each read was known, an estimate of these prior probabilities could be obtained by observing the fraction of reads generated by each reference. However, each read is essentially split among several possible 'read-generating' reference sequences probabilistically from the previous E-step. Thus, we compute:

where *J *is the total number of reads with mappings.

### Algorithm initialization and termination

The EM algorithm performs best and avoids local maxima when initialized with reasonable parameters. Therefore, we initialize EMIRGE by first choosing the single best reference sequence for each read, as reported by the mapping software. If a read maps equally to two or more sequences, a reference sequence is chosen at random. When paired reads are available, the reads are mapped together and the probabilities are computed as if a single longer read was used. We then assume for each read-reference pair and begin an M-step. We terminate the algorithm when no further changes are made to the nucleotide sequence of the reference SSU genes, at which time the sequence abundances have also stabilized (Figure 2a, c).

### Assessing algorithm performance on simulated microbial communities

**Figure 3:**
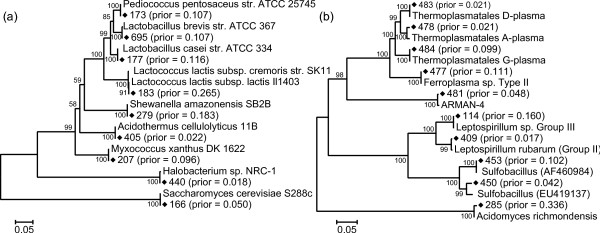
**Community composition is captured by correctly reconstructed full-length SSU sequences**. **(a, b) **Phylogenetic trees showing algorithm-reconstructed sequences (black diamonds) and their best blast hits, for both the simulated simple **(a) **and natural **(b) **microbial communities. Reconstructed sequences are presented with their (arbitrary) algorithm-assigned identifier and their prior probability, which serves as an abundance estimate, after the final round. All reconstructed sequences match to the expected organism in the simulated community (a), and all but two sequences match to metagenomic contigs assembled from traditional Sanger sequencing in the natural community (b). The two novel sulfobacilli in the natural community are presented with their closest blast hit in GenBank. Units are base substitutions per site, and bootstrap values ≥ 50 are shown at the branches.

**Figure 4:**
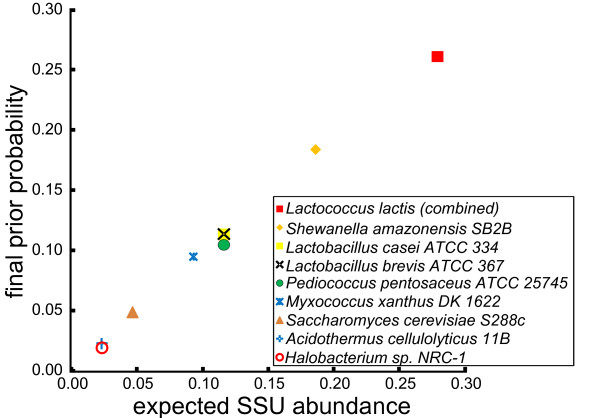
**SSU abundance estimates are accurate**. For the nine most abundant reconstructed sequences in the simulated simple community, the final prior probability estimated by EMIRGE is plotted against the expected SSU abundances from the associated community members. The algorithm recovers the expected abundances excellently (Pearson *ρ *= 0.998, *P*-value = 8.5e-10).

To test the ability of EMIRGE to reconstruct correct SSU gene sequences and abundances from metagenomic data, we first simulated realistic error-containing Illumina reads from the simple microbial community constructed *in silico *(see Materials and methods). After combining SSU genes from the same organism (generating composite sequences in cases where multicopy genes were not identical), and combining two *Lactococcus lactis *subspecies, which share near identical SSU sequences (> 99% identity), the nine composite SSU genes range in abundance from 2.3% to 26.1%. To further challenge our algorithm, we also introduced random mutations in 10% of the bases in each SSU sequence in the starting reference database. We processed the simulated reads with EMIRGE and examined the nine most abundant reconstructed SSU sequences that emerged from the starting database of approximately 125,000 sequences (Additional file 1). These nine sequences, which together represented 96% of the summed prior probabilities, accurately recover each of the expected SSU sequences (Figure 3a). In addition, the observed abundances match the expected abundances excellently (Pearson *ρ *= 0.998, *P*-value = 8.5e-10; Figure 4). Thus, on simulated but realistic data, our method accurately reconstructs both community SSU gene sequences and their abundances.

**Figure 5:**
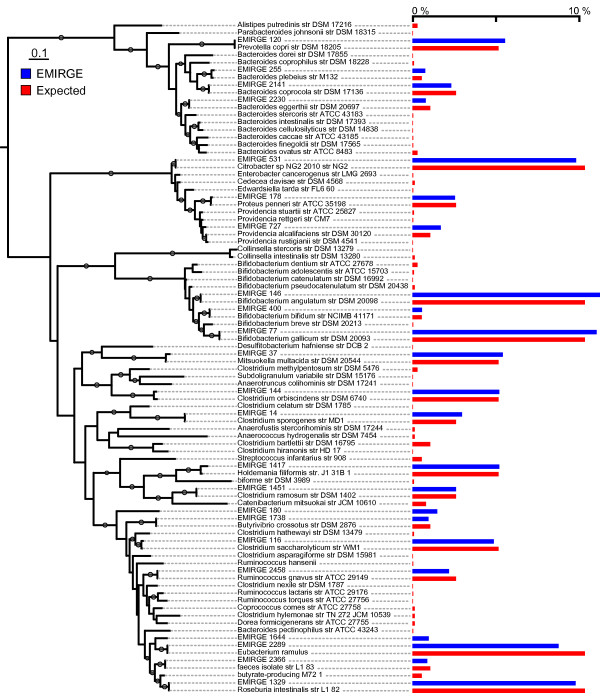
**Accurate SSU sequences and abundance estimates are recovered by EMIRGE for a complex microbial community**. Using reads from the complex simulated community, full-length SSU genes reconstructed by EMIRGE with estimated abundances of > 0.5% were aligned and placed in a phylogenetic tree with the expected community members. Estimated EMIRGE sequences and relative abundances (blue) correspond in most cases to expected sequences and expected abundances (red). Grey circles on branches indicate bootstrap values > 80.

**Figure 6:**
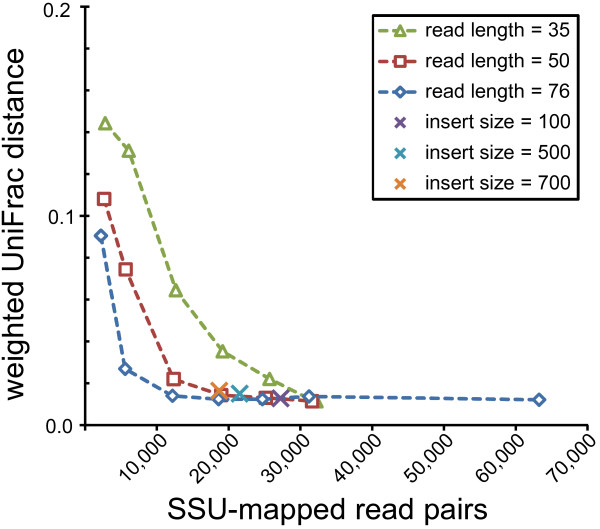
**Effect of sequencing library characteristics on EMIRGE performance**. The effects of sequencing effort (x axis), read length, and insert size were evaluated by running EMIRGE on the complex community with varying input. Reconstructed communities were compared to the expected community with the weighted UniFrac distance metric [30]. For the varying insert size experiment, a single sequencing effort was chosen (76-bp read length; 80,000 genomic reads; see Materials and methods).

To gauge how EMIRGE performs on more complex communities, we next simulated reads from a mock community of 67 human gut microbes [28]. The community reconstructed from the full-length SSU sequences reported by EMIRGE was highly similar to the expected community (Figure 5; Additional file 2). We attempted to quantify this similarity; using the weighted UniFrac statistic [30], the EMIRGE and expected communities were not distinguishable (weighted UniFrac distance = 0.0124, *P*-value < 0.01). The EMIRGE community was more similar to the expected community when the input reads were longer or sequencing effort was higher. However, even with few paired SSU reads of full length, or with enough reads as short as 36 bp, the community structure could be recovered correctly (Figure 6). Insert size of the sequencing library had little effect on the ability of EMIRGE to recover the expected community.

We also attempted to evaluate how well the short SSU fragments produced by *de novo *assembly could reconstruct the structure of a microbial community. Assembly produced many more SSU gene fragments than expected for each of the simulated communities, though many of these could be classified to a specific genus (Table 1). We used BLAST [31] to search for the closest known full-length SSU sequence to these short assembled fragments. The best hits were clustered at 97% identity, and each cluster was given an abundance relative to the average k-mer coverage of the matching SSU fragments. The resulting phylogenies produced weighted UniFrac distances to the expected communities that were roughly an order of magnitude larger than the distances produced by EMIRGE (Table 1).

### Assessing algorithm performance on a natural microbial community

**Figure 7:**
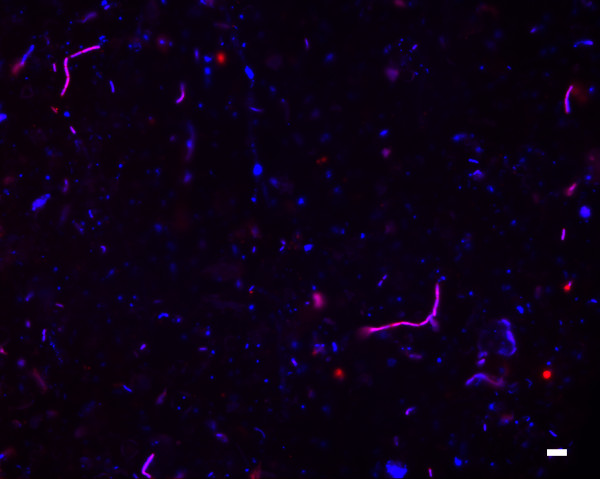
**Validation of the presence of *Sulfobacillus *in the natural community**. Fluorescent *in situ *hybridization with a *Sulfobacillus*-specific probe (red) shows that *Sulfobacillus *is present in the natural community, as predicted by EMIRGE. The generic DNA stain DAPI is shown in blue, and *Sulfobacillus *cells with both the specific probe and DAPI staining appear purple. Scale bar: 5 μm.

Next, we applied EMIRGE to the model natural microbial community from acid mine drainage [23]. We sequenced a single lane of a 76-bp paired-end Illumina library. As with the simulated communities, we mutated 10% of the bases in each SSU sequence in the starting reference database to test if new taxa can be discovered. We then ran our algorithm until convergence and examined the most probable reconstructed SSU sequences with abundance ≥ 1% (Figure 2d; Additional file 3). Because this particular acid mine drainage community is closely related to communities that have been extensively studied via metagenomics, proteomics, and traditional clone libraries, we were able to validate nearly all of the reconstructed SSU sequences by comparing them to known metagenomic contigs (Figure 3b). In addition to obtaining correct species-level SSU sequences from the typical microbial members that dominate this community, we also discovered novel *Sulfobacillus*-like SSU sequences not found in previous metagenomic assemblies whose closest sequence homology is to SSU clones recovered from ore-processing environments [Genbank: EU419137 and AF460984]. We confirmed the presence of *Sulfobacillus *in our real community by fluorescent *in situ *hybridization with a probe designed specifically to this genus (Figure 7). The presence of the *Sulfobacillus *genus could also be detected based on short SSU fragments produced by the *de novo *assembly. However, these fragments were not long enough to give the species-level assignment of the EMIRGE *Sulfobacillus *sequences. We identified 37 different short fragments (70 to 243 bp) in the assembly that were assigned by the RDP Classifier [32] to the *Sulfobacillus *genus. Thus, our method recovers known and novel full-length SSU genes from species residing in a natural microbial community.

## Discussion

When characterizing microbial communities, a critical goal for metagenomic data analysis is recovery of a collection of full-length SSU sequences, each of which represents an operational taxonomic unit. However, when short read metagenomic datasets sampling coexisting organisms are assembled, the SSU genes tend to be highly fragmented and misassembled (Figure 1). The resulting short contigs are often composite sequences, not representative of any individual taxon present. Complexity arises because sequences in highly homologous regions co-assemble, while assembly paths diverge where sequence variation exceeds some defined threshold. Identification of the appropriate path is confounded when reads are shorter than the distance between low variation regions. EMIRGE solves these problems by avoiding traditional assembly altogether, probabilistically reconstructing full-length SSU gene sequences from metagenomic datasets. To our knowledge, this is the first report of successful full-length SSU reconstruction from short read metagenomic sequencing data. The method also accurately estimates relative abundances of SSU sequences from each organism type (Figures 4 and 5). Of course, like all approaches relying on the SSU rRNA gene for quantification, gene copy number can confound abundance estimates [33].

Full-length genes provide more complete taxonomic information than prior tag-sequencing approaches that have sequenced PCR-amplified short hypervariable regions (typically < 200 bp) [3-7]. Tag sequencing is subject to potential primer bias [16] and ultimately relies on underlying phylogenies built from full-length genes. Although the phylogenetic information contained in hypervariable region tags is generally concordant with information contained in full-length sequences at higher taxonomic levels [3,5], careful screening of read quality is necessary to avoid overestimating community diversity [34,35]. Others have argued that full-length Sanger sequencing of traditional clone libraries remains the only way to adequately construct the phylogeny of life [36]. The method presented here offers a cost-effective alternative for reconstructing accurate full-length sequences. The increased read depth underlying each reconstructed sequence ensures that no single read or its potential sequencing errors inflate diversity estimates.

One key to the success of EMIRGE lies in the iterative approach encapsulated in the EM algorithm (Figure 2). The deep coverage of Illumina sequencing has been used before to iteratively map and correct whole genome consensus sequences from a single species [37], or a population of closely related strains [38]. Our approach differs in both the end goal and the statistical approach taken: the EM algorithm models a true population of SSU genes, and constructs only probabilistic descriptions of both the SSU sequences and their underlying abundances. Reference sequences that show evidence (in the form of multiple ambiguous base probabilities above some threshold) of multiple strains are split and allowed to evolve separately, rather than forcing reads into a single composite sequence. Even when iteration ends, base probabilities in the reconstructed SSU sequence can reveal likely single nucleotide polymorphisms in closely related but distinct subspecies in the community (Figure 1c).

Also central to our approach is the handling of uncertainty by the EM algorithm. This algorithm has a wide variety of applications in high-throughput biological experimentation, which often must deal with hidden data [39]. Here, rather than try to make a definitive statement about every read, ambiguity created by short reads and high homology within the SSU gene is dealt with probabilistically. Thus, evidence for the sequence and abundance of a particular SSU gene also accumulates probabilistically, with more evidence accumulating from more probable read mappings in each iteration. The result is a set of SSU sequences in which each reconstructed nucleotide has a confidence estimate based on its final probability. The approach was validated by recovery of the anticipated set of sequences from both simulated and natural community datasets (Figures 3 and 5) at a level of taxonomic resolution typically used to define operational taxonomic units (OTUs; 97% identity). The algorithm can be tuned to higher levels of stringency (for example, 99%) if desired, an important feature given the diversity of genomes and metabolisms for organisms with similar or even identical SSU sequences.

The benefit of the probabilistic EM strategy is demonstrated by the accuracy of the SSU reconstructions obtained, even when 10% of nucleotide positions were mutated in the underlying SSU database. This robustness of the method to database error means that new taxa can be discovered. For example, we were able to recover a novel *Sulfobacillus *SSU gene not identified in previous metagenomic and PCR-based analyses of similar biofilm communities. This gene shared only 88% identity with the closest sequence in the starting reference database. We have also applied the EMIRGE method to the description of thermophilic bacterial consortia adapted to grow on switchgrass (JM Gladden *et al*.: Community dynamics and glycoside hydrolase activities of thermophilic bacterial consortia adapted to switchgrass, submitted). The method recovered full-length SSU genes that corresponded closely to phylogenetic identifications derived from amplicon-based pyrotag sequencing for these communities, and the EMIRGE prior probabilities showed general concordance with the abundance estimates made by pyrotag sequencing.

Our implementation runs overnight on standard hardware available to most labs studying microbial ecology (see Materials and methods). However, the method makes assumptions and choices for computational speed that could be improved upon. In its current form, for example, we have chosen a read mapper [19] that, while extremely fast, is blind to insertions and deletions (indels). We find that the main effect of this choice is the occasional presence of small indel errors in the reconstructed sequence. In practice, these rare indels have little effect on taxonomic assignment. Future EMIRGE implementations will incorporate a method to handle indels. This will allow for extension of EMIRGE to genes with higher levels of sequence divergence and make it useful for reconstruction of full-length SSU genes from metagenomes sequenced with Roche 454 technology (which is prone to indel errors at homopolymer runs [34]).

Additionally, although the method adequately corrects nucleotide errors in the reference database, it is possible that chimeric database sequences could carry over into SSU reconstructions, if reads map across the full length of the chimera. None of the EMIRGE-generated sequences reported here were identified as chimeric (data not shown); however, we have documented at least one very low abundance chimera (below the reporting threshold) in the natural community that evolved from a chimeric database sequence. Strict database pre-screening should eliminate this potential problem. EMIRGE-generated sequences would benefit from the same downstream quality control applied to traditional clone libraries.

As it is described here, EMIRGE does not suffer from potential primer bias introduced by so-called 'universal' primers [16], allowing for discovery of novel species that may not have canonical primer binding sites [18]. Like other methods that use next generation sequencing technologies [3-5], the method also removes potential cloning bias introduced with Sanger sequencing. However, it may be subjected to other biases associated with new technologies - for example, the under-representation of sequences at the extremes of GC content [40].

In its current form, the analysis relies upon only the small fraction (< 0.2% here) of reads that derive from SSU genes. However, the EMIRGE algorithm could easily be applied to full-length SSU amplicon datasets, enabling confident reconstruction of full-length SSU genes from extremely low-abundance organisms. We focused here on demonstrating the accuracy of the method for reconstruction of SSU sequences for OTUs representing ≥ 1% of the population. If the method scales linearly, we can expect to recover accurate sequences and abundances from SSU genes from organisms representing just 0.002% of similar populations with a single lane of Illumina amplicon sequencing.

## Conclusions

The method reported here, EMIRGE, reconstructs full-length SSU sequences from metagenomic data from a microbial community of interest, accurate to the species level. In addition, the method also provides accurate SSU sequence abundance estimates. EMIRGE is robust to errors and omissions in the reference database, and is broadly applicable to any dataset produced with short read sequencing technology. An open-source implementation of the algorithm is freely available [41]. We expect that application of the method, especially with very deep sequencing, will provide new insights into fine details of changing microbial community structure.

## Materials and methods

### Sample collection, short-read sequencing and assembly of the natural microbial community

Fungal streamer biofilms were collected in February 2008 from the 5-way site of the Richmond Mine at Iron Mountain, California [23]. The pH of the acid mine drainage the biomass was sampled from was 0.98, and the temperature was 38°C.

For genomic DNA extraction, aliquots of 4 to 5 ml of frozen biofilm were thawed in an equal volume of 4°C 0.9% NaCl, pH 1.0. Biofilm was homogenized with a pipette tip, and then pelleted by centrifugation at 7,000 × g for 5 minutes at 4°C. The supernatant was removed, and 4 ml of 4°C phosphate buffered saline, pH 7.0, was added to the cell pellet. The cells were passed several times through a 16 G needle to further homogenize the biofilm, and again pelleted at 7,000 × g for 5 minutes at 4°C. The supernatant was removed, and the cell pellet was added to a sterile, pre-chilled mortar and ground to a fine powder in liquid nitrogen. This frozen powder was stored in liquid nitrogen for further processing. Approximately 50 mg aliquots of frozen, ground powder were added to tubes with pre-warmed (65°C) lysis solution from the PowerSoil DNA Isolation Kit (MoBio Laboratories, Carlsbad, CA, USA). This mixture was incubated with shaking at 120 rpm for 10 minutes at 65°C, with brief vortexing every 2 minutes to resuspend the powder in the lysis buffer. The mixture was then bead beat for 30 s at 5 m/s in the provided tubes, and the manufacturer's protocol was followed for DNA extraction and cleanup. DNA was eluted in TE buffer, and aliquots were pooled and DNA precipitated with 2 volumes of EtOH before resuspending in TE buffer for library preparation.

Illumina library preparation and sequencing followed standard protocols developed at the Joint Genome Institute [42]. Briefly, genomic DNA was sheared by nebulization, and sheared fragments were end-repaired and phosporylated. Blunt-end fragments were A-tailed, and sequencing adapters were ligated to the fragments. Fragments with an insert size of around 200 bp were gel-extracted and enriched with 12 cylces of PCR before library quantification and validation. Hybridization of the library to the flow cell and bridge amplification was performed to generate clusters, and paired-end reads of 76 cycles were collected.

A single flow cell lane was used to obtain 38.6e6 paired-end 76-bp reads (2.9 Gbp). The raw reads have been deposited in the NCBI Sequence Read Archive under accession [SRA:SRR191843]. These reads were used as input to the assembler Velvet [29]. For the natural and the simulated communities, the VelvetOptimiser script was run with default parameters to choose assembly k-mer and coverage parameters that led to optimal assembly (k = 49 for the natural community, k = 61 for the simple simulated community, k = 37 for the complex community). The LastGraph file produced by Velvet was used for the generation of Figure 1.

### Simulating short read sequencing reads for simulated microbial communities

For the simple simulated community, we reconstructed reads from the ten evenly distributed genomes used in the *in vitro *community of Morgan *et al*. [27]. For the complex community, we first downloaded the SSU sequences for organisms in the 'uneven 1' mock community from Turnbaugh *et al*. [28]. Because not all of these organisms have available genome sequences, we padded each SSU sequence with 1,000 random bases before simulating reads. This likely makes the *de novo *assembly problem (though not EMIRGE's method) easier than can be expected in a real community, as there are unlikely to be shared k-mers between taxa in these random padding sequences. We used the wgsim program [43] to simulate 60e6 paired-end, 76-bp, error-free reads from the genomes for the simple community, with an insert length mean of 200 bp and a standard deviation of 25 bp. We simulated reads with varying coverage depth, insert size, and read length for the complex community. Unless otherwise specified, we used a data set with 80,000 paired, 76-bp reads with an insert size mean and standard deviation of 300 ± 30 bp. When simulating other insert sizes, a standard deviation of 10% was used. Many of these simulated reads fell in the padded genome sequence outside of the SSU genes. We assigned quality score vectors to each read by sampling at random from 1 million real quality value vectors from the real microbial community reads, and made mutations in all simulated reads with the probabilities specified by the assigned quality scores. To calculate the expected abundances of SSU rRNA genes in the simple simulated community, we divided the copy number of the SSU gene for any given genome by the sum of copy numbers for all SSU genes in the community. For the complex community, we used the expected abundances given by Turnbaugh *et al*. [28]. Both simulated datasets are available [44].

### Evaluation of SSU gene fragments produced by *de novo *assembly

Contigs from *de novo *assembly were searched with BLAST [31] against the Silva SSU database, and contigs with an e-value ≤ 1e-10 were identified as SSU-fragment-containing. These fragments were classified at the genus level using the RDP classifier [32] if the classification had a bootstrap value > 50%. Best BLAST hits to the fragments were clustered at 97% identity using UCLUST [45], and relative abundances for each cluster representative were calculated based on the average k-mer coverage, as reported by Velvet, for each of the fragments hitting a sequence in that cluster. We note that this strategy will not allow the discovery of novel sequences as EMIRGE will; however, for the two simulated datasets all the expected sequences were in the search database.

### Implementation details

The reference SSU database was built by first removing sequences < 1,200 bp or > 1,900 bp from the Silva [15] SSU reference 102, and then clustering the remaining sequences at 97% identity with UCLUST [45]. In order to evaluate the ability of EMIRGE to recover novel sequences, for each database sequence, 10% of the sites were chosen at random and mutated to a different base, also chosen at random. For read mapping, we used the short read mapper bowtie [19] in paired-end mode. For initiation, bowtie reported a single best alignment, allowing up to three mismatches in a 20-bp seed, and a maximum sum of quality values in mismatched bases of 300. The minimum and maximum insert size allowed was set to ± 3 times the expected standard deviation around the expected median insert size. For subsequent iterations, bowtie used the same parameters, but reported all mappings in the top strata. Prior to running EMIRGE, reads were trimmed from the 3' end to remove bases with quality scores of 2 or lower, and paired reads were kept if both reads were at least 60 bp long after trimming. For data sets with shorter read lengths, no trimming was done.

At each iteration, SSU sequences were merged into one sequence if the identity of non-gapped positions in a global alignment was greater than 97%. A single SSU sequence (and its prior probability) was divided into two sequences if the second most probable base in more than 4% of all positions had a probability greater than 10%. In this way, sequences that evolved over iterations to be the same were merged, and sequences with evidence from the reads for multiple OTUs were duplicated and allowed to evolve as separate OTUs in future iterations.

EMIRGE was implemented in python. Forty iterations of EMIRGE for the natural community took 6.8 hours on eight Intel Xeon 2.0 GHz cores, with a maximum memory footprint of 900 MB. The read mapping steps accounted for 91% of the run time and is parallelizable. Thus, run time decreases approximately linearly with the number of processor cores available.

### Fluorescent *in situ *hybridization microscopy

Cell fixation and epifluorescent microscopy were performed as described previously [46], except that cells were fixed after thawing from storage at -80°C. We developed a probe for a broader specificity of *Sulfobacillus *spp. from Bond *et al*. [47], SUL230 (5'-GGRGCUCGCGGCCCAUUA-3'), and all cells were counterstained with DAPI. Probe stringency was maintained by hybridization at 46°C with 30% formamide.

### Phylogenetic tree construction and evaluation of community structure

The nine (for the simple simulated community) and ten (for the real community) most abundant SSU sequences recovered by the algorithm were used for phylogenetic tree construction. For the simple simulated community, these sequences were aligned with default parameters with muscle [48] with the known SSU genes. For the real community, we blasted reconstructed SSU sequences against previously assembled contigs from the same environment [GenBank:ADCE01000000, GenBank:ADHF00000000.1, GenBank:ABOZ00000000.1, GenBank:ACXJ00000000.1, GenBank:ACVJ01000000, GenBank:ACXK01000000, GenBank:ACXL01000000, GenBank:ACXM01000000, GenBank:ACNP01000000, GenBank:AAWO01000000, GenBank:AADL01000000], and the Silva database, and included the best hit for each reconstructed sequence in the resulting multiple sequence alignment. These alignments were imported into MEGA [49], and neighbor joining trees with 500 bootstrap replicates were built using distances derived with the maximum composite likelihood method. Only positions in the alignments without gaps were used to construct the trees.

For the complex community, we aligned EMIRGE-generated SSU sequences with abundance estimates > 0.5% with the sequences from the known community with muscle, and built maximum likelihood trees with RAxML [50] using the GTRGAMMA model. We used these, and analogously built trees for input reads with varying library parameters, to measure the weighted UniFrac distance [30] between the reconstructed and known communities using the UniFrac website [51]. For comparisons with the communities produced by clustering the best hits to *de novo *assembly fragments, a single alignment was built for the three communities with expected, EMIRGE-produced, and assembly-fragment-best hit sequences. A tree was built using FastTree [52], and weighted UniFrac values were computed for each pair of communities using this single phylogeny. Figure 5 was constructed using iTOL [53].

## Abbreviations

Bp: base pair; DAPI: 4',6-diamidino-2-phenylindole; EM: expectation maximization; EMIRGE: expectation maximization iterative reconstruction of genes from the environment; E-step: expectation step; M-step: maximization step; OUT: operational taxonomic unit; PCR: polymerase chain reaction; SSU: ribosomal small subunit.

## Competing interests

The authors declare that they have no competing interests.

## Authors' contributions

CSM, BJB, SWS, and JFB designed the study. CSM performed the experiments, and designed and implemented the algorithm. BJB designed the FISH probe. CSM and JFB wrote the manuscript. All authors analyzed the data, and read and approved the final manuscript.

## Supplementary Material

Additional file 1 **FASTA formatted file containing EMIRGE-reconstructed sequences from the simple simulated microbial community**. Click here for file

Additional file 2 **FASTA-formatted file containing EMIRGE-reconstructed sequences from the complex simulated microbial community**. Click here for file

Additional file 3 **FASTA-formatted file containing EMIRGE-reconstructed sequences from the natural microbial community**. Click here for file
